# *TRIM39-RPP21* Variants (∆19InsCCC) Are Not Associated with Juvenile Idiopathic Epilepsy in Egyptian Arabian Horses

**DOI:** 10.3390/genes10100816

**Published:** 2019-10-16

**Authors:** Victor N. Rivas, Monica Aleman, Janel A. Peterson, Anna R. Dahlgren, Erin N. Hales, Carrie J. Finno

**Affiliations:** 1 Department of Population, Health and Reproduction, University of California, Davis, CA 95616, USAjanel.peterson@bcm.edu (J.A.P.); adahlgren@ucdavis.edu (A.R.D.); 2 Department of Veterinary Medicine and Epidemiology, University of California, Davis, CA 95616, USA; mraleman@ucdavis.edu

**Keywords:** convulsion, electroencephalogram, equine, seizure

## Abstract

Juvenile idiopathic epilepsy (JIE) is an inherited disease characterized by recurrent seizures during the first year of life in Egyptian Arabian horses. Definitive diagnosis requires an electroencephalogram (EEG) performed by a veterinary specialist. A recent study has suggested that a 19 base-pair deletion, along with a triple-C insertion, in intron five of twelve (∆19InsCCC; chr20:29542397-29542425: GTTCAGGGGACCACATGGCTCTCTATAGA>TATCTTAAGACCC) of the *Tripartite Motif-Containing 39-Ribonuclease p/mrp 21kDa Subunit* (*TRIM39-RPP21*) gene is associated with JIE. To confirm this association, a new sample set consisting of nine EEG-phenotyped affected and nine unaffected Egyptian Arabian horses were genotyped using Sanger sequencing. There was no significant genotypic (*P* = 1.00) or allelic (*P* = 0.31) association with the ∆19InsCCC variant and JIE status. The previously reported markers in *TRIM39-RPPB1* are therefore not associated with JIE in well-phenotyped samples. The ∆19InsCCC variant is a common variant that happens to be positioned in a highly polymorphic region in the Arabian breed.

## 1. Introduction

Juvenile idiopathic epilepsy (JIE) is an inherited disease characterized by recurrent seizures during the first year of life in Egyptian Arabian horses. Recurrent seizures can lead to complications, including head injury and aspiration pneumonia. After the first year of life, seizure activity ceases [1]. There are no known environmental factors linked to the clinical presentations of JIE [2]. A definitive diagnosis of JIE requires an electroencephalogram (EEG) to confirm the presence of paroxysmal activity (sharp waves and spikes) supportive of seizures during clinical manifestations of epilepsy [3,4,5]. Due to the similarity with other genetically associated epileptic disorders in humans [6] and its occurrence in specifically the Egyptian Arabian breed, veterinary researchers postulate that the disease may have a genetic basis [1,7,8]. Interpretation of pedigree data suggests that JIE may be heritable with an autosomal dominant mode of inheritance (MOI) (unpublished data). Recently, a study was published documenting an intronic 19 base-pair deletion coupled with a triple-C insertion (∆19InsCCC; chr20:29542397-29542425: GTTCAGGGGACCACATGGCTCTCTATAGA>TATCTTAAGACCC) in the *TRIM39-RPP21* gene as associated with JIE [9]. The aim of this study was to confirm this association in a group of EEG-confirmed affected and veterinary-reported unaffected Egyptian Arabian foals. 

## 2. Materials and Methods

Eighteen Egyptian Arabian horses were used in this study (nine affected and nine unaffected horses). Affected horses were phenotyped via an EEG examination by a board-certified veterinary neurologist (M.A.) as previously described [1]. Unaffected horses were determined to have no history of seizures throughout the first year of life by both owner and veterinary reports. Genomic DNA of all horses was isolated from EDTA samples using a Wizard^®^ Blood DNA extraction Kit (Promega, Madison, WI, USA). DNA samples were quantified with a QIAxpert (QIAGEN, Hilden, Germany) and diluted to 50 ng/μl. Genotyping at the ∆19InsCCC position was carried out using the previously reported primer set [9]. DNA was amplified using polymerase chain reaction (PCR) and product sizes were confirmed on a 1% agarose gel. As some reactions consistently showed secondary amplification products, two new primer sets were constructed with the use of Primer3Plus [10] and UCSC In-Silico PCR [11] programs (Table S1). Individual reactions were run at the following settings: 95 °C for 15 m, followed by 35 cycles of 95 °C for 30 s, 60 °C for 30 s, 72 °C for 30 s, and 72 °C for 5 m. Due to the polymorphic nature of this region, reactions were set up in a range of annealing temperatures (58–67°C). An ExoSAP-IT PCR Product Cleanup Kit (Thermo Fisher Scientific, Waltham, MA, USA) was used on all amplified samples, and amplicons were sequenced at the UCDNA sequencing facility (Davis, CA, USA). Raw .abi sequencing files were viewed using SEQUENCHER^®^ (Gene Codes, Ann Arbor, MI, USA), and genotypes were manually determined. Association testing with the JIE phenotype was performed using genotypic and allelic-based Fisher’s exact tests, with *P* < 0.05. The presence of ∆19InsCCC indels were further screened using data from Arabian horses in NCBI’s Sequence Read Archive (SRA) database (https://www.ncbi.nlm.nih.gov/sra).

## 3. Results

With the use of three primer sets, all samples were successfully genotyped. The region containing the ∆19InsCCC variant is highly polymorphic; the presence of additional single nucleotide polymorphisms (SNPs) and/or indels at both 5’ and 3’ of the ∆19InsCCC boundary made construction of specific primers difficult (Figure S1). Both JIE-affected and unaffected horses had varying numbers of additional variants in this region (Table S2). 

Of the nine unaffected horses, five were homozygous wild-type, two were homozygous for the ∆19InsCCC variant, and two were heterozygous. Of the nine EEG-confirmed affected horses, four were wild-type, and the remaining five were homozygous for the ∆19InsCCC variant (Table 1). A genotypic Fisher’s exact test, assuming an autosomal dominant MOI for the disease, revealed no association with the disease (*P* = 1.00). An allelic Fisher’s exact test also showed no statistically significant association between the ∆19InsCCC variant and the disease (*P* = 0.31). Within NCBI’s SRA database of 384 mapped equine genomes, only three (ERX2235970, ERX2235963, and ERX1598843) were Arabian horses (lineages unknown), and all were homozygous wild-type at the ∆19InsCCC locus.

Previous work postulated that the ∆19InsCCC variant was positioned within an unannotated exon, rather than in an intronic region, and therefore led to changes in the coding sequence [9]. To test this hypothesis, EquCab2.0 alignment files from the Functional Annotation of the Animal Genome (FAANG) project were viewed at the ∆19InsCCC locus using the Integrative Genomics Viewer (IGV) [12]. FAANG RNA-seq (https://www.ebi.ac.uk/ena/data/view/ERA148755) data revealed no expression at the ∆19InsCCC position in any sequenced neural tissues (cerebellar vermis, frontal cortex, hypothalamus, lateral cerebellum, occipital cortex, parietal cortex, and retina).

## 4. Discussion

The data gathered in this study refutes the genetic association with the previously described ∆19InsCCC variant and JIE. As the original study [9] defining this association was unable to confidently phenotype unaffected Arabian horses, this initial association may have been a false positive due to the prevalence of this variant in the Egyptian Arabian breed. Horses in the previous study were diagnosed with JIE based on veterinary observation alone, as EEG testing was not performed. Additionally, only one horse in the previous study could be confidently classified as a control [9]. In our study, JIE affected horses were phenotyped via EEG, and previous seizure events and unexplained trauma during the first year of life were well documented on all affected and unaffected horses. Importantly, four out of nine of our EEG-confirmed JIE-affected foals were wild-type for the putative variant, making it highly unlikely that the ∆19InsCCC variant within *TRIM39-RPP1* is causative for JIE. This is likely a common variant in the Arabian breed; however, genotyping of a random sample of Arabian horses is necessary to confirm this theory. Additionally, with FAANG horse transcriptome data, we were able to determine that the ∆19InsCCC is within an intron of *TRIM39-RPP1*, as data revealed no expression at the ∆19InsCCC position in any sequenced neural tissues from adult horses. However, the possibility remains that expression of this region may be specific to a certain developmental time point.

The region surrounding the ∆19InsCCC variant within *TRIM39-RPP1* was particularly challenging to genotype due to a high degree of polymorphism 3’ of the indel (Table S2) as well as some additional variants 5’ of the indel. In particular, genotyping of heterozygotes led to mixed peaks shortly after the start of the indel. With such a high degree of polymorphism within this region, it is unlikely that this region would reliably contribute to the JIE phenotype.

In conclusion, due to the high frequency of the ∆19InsCCC variant in both the JIE-affected and unaffected horses (*q* = 0.44), we exclude these indels to be causative of JIE. Further genetic evaluation of JIE, with genome-wide association studies, is required using EEG-confirmed samples. 

## Figures and Tables

**Table 1:** Egyptian Arabian population (n=18) ∆19InsCCC genotypes.

Status	Homozygous ∆19InsCCC	Heterozygous	Homozygous Wild-Type	Total
Unaffected JIE	2	2	5	9
Affected JIE	5	0	4	9
Total	7	2	9	18
*P*<0.05, Genotypic Fisher’s *P* = 1.00, Allelic Fisher’s *P* = 0.31				

## Supplementary Materials

The following is available online at https://www.mdpi.com/2073-4425/10/10/816/s1, Table S1: Primers for Sanger sequencing of the ∆19InsCCC region in *TRIM39-RPP2*, Table S2: Egyptian Arabian population (n = 18) ∆19InsCCC genotypes and additional variants, Figure S1: Variants of 3’ within the *TRIM39-RPP1* sequence, Figure S1: An example chromatogram from an affected (JIE4) and unaffected (JIE18) Egyptian Arabian foal within the ∆19InsCCC region in *TRIM39-RPP2*. There is extensive variation 3’ of the ∆19InsCCC region, as depicted by stars (SNPs) and triangles (indels).
