# Supplementary material for: TRIM39-RPP21 Variants (∆19InsCCC) Are Not Associated with Juvenile Idiopathic Epilepsy in Egyptian Arabian Horses
Source: Genes (Basel). 2019 Oct 16;10(10):816. doi: 10.3390/genes10100816 (PMC6826448; doi:10.3390/genes10100816)
Supplement: Supplementary file 1 [file genes-10-00816-s001.zip › genes-610877 supplementary/genes-610877-supplementary-english.docx]

| **Supplementary Table 1.** Primers for Sanger sequencing of the **∆**19InsCCC region in *TRIM39-RPP21*. | | |  |
| --- | --- | --- | --- |
| Primer Name (F/R) | | Primer Sequence (F/R) | Amplicon Size |
| *Original_TRIM39_F / Original_TRIM39_R* | | 5’GATGAACTATAACAGTATCCCCAGA3’ / 5’ATACTGAAGTCACCTCCATGGTCT3’ | 238 bp |
| *New1_TRIM39_F / New1_TRIM39_R* | | 5’AGATCTAGAACTTTTGGGAGGAG3’ / 5’TAAGGCTGCTCCCTTTGAG3’ | 972 bp |
| *New2_TRIM39_F / New2_TRIM39_R* | | 5’TCCTTGGTGAGGAAGATACCA3’ / 5’CCTCCATGGTCTTCACCTTC3’ | 395 bp |

| **Supplementary Table 2.**  Egyptian Arabian population (n=18) ∆19InsCCC genotypes and additional variants. | | | | |
| --- | --- | --- | --- | --- |
| Horse ID | Status | Genotype | 5' Variants | 3' Variants |
| JIE1 | Unaffected | ∆19InsCCC/N | Deletions | - |
| JIE2 | Unaffected | N/N | SNPs | Insertions & Deletions |
| JIE3 | Unaffected | ∆19InsCCC/∆19InsCCC | SNPs | SNPs, Insertions, & Deletions |
| JIE4 | Unaffected | ∆19InsCCC/∆19InsCCC | SNPs | SNPs, Insertions, & Deletions |
| JIE5 | Unaffected | ∆19InsCCC/N | None | - |
| JIE6 | Unaffected | N/N | SNPs | Insertions & Deletions |
| JIE7 | Unaffected | N/N | None | Deletions |
| JIE8 | Unaffected | N/N | SNPs & Deletions | Insertions & Deletions |
| JIE9 | Unaffected | N/N | SNPs | Insertions & Deletions |
| JIE10 | Affected | ∆19InsCCC/∆19InsCCC | SNPs | SNPs & Deletions |
| JIE11 | Affected | N/N | None | SNPs, Insertions, & Deletions |
| JIE12 | Affected | N/N | SNPs | SNPs, Insertions, & Deletions |
| JIE13 | Affected | ∆19InsCCC/∆19InsCCC | SNPs & Deletions | SNPs & Deletions |
| JIE14 | Affected | ∆19InsCCC/∆19InsCCC | SNPs | SNPs & Insertions |
| JIE15 | Affected | N/N | SNPs & Deletions | SNPs, Insertions, & Deletions |
| JIE16 | Affected | N/N | SNPs | SNPs, Insertions, & Deletions |
| JIE17 | Affected | ∆19InsCCC/∆19InsCCC | SNPs & Insertions | SNPs & Deletions |
| JIE18 | Affected | ∆19InsCCC/∆19InsCCC | SNPs & Deletions | SNPs, Insertions, & Deletions |
