# Supplementary figures and images for: TRIM39-RPP21 Variants (∆19InsCCC) Are Not Associated with Juvenile Idiopathic Epilepsy in Egyptian Arabian Horses
Source: Genes (Basel). 2019 Oct 16;10(10):816. doi: 10.3390/genes10100816 (PMC6826448; doi:10.3390/genes10100816)

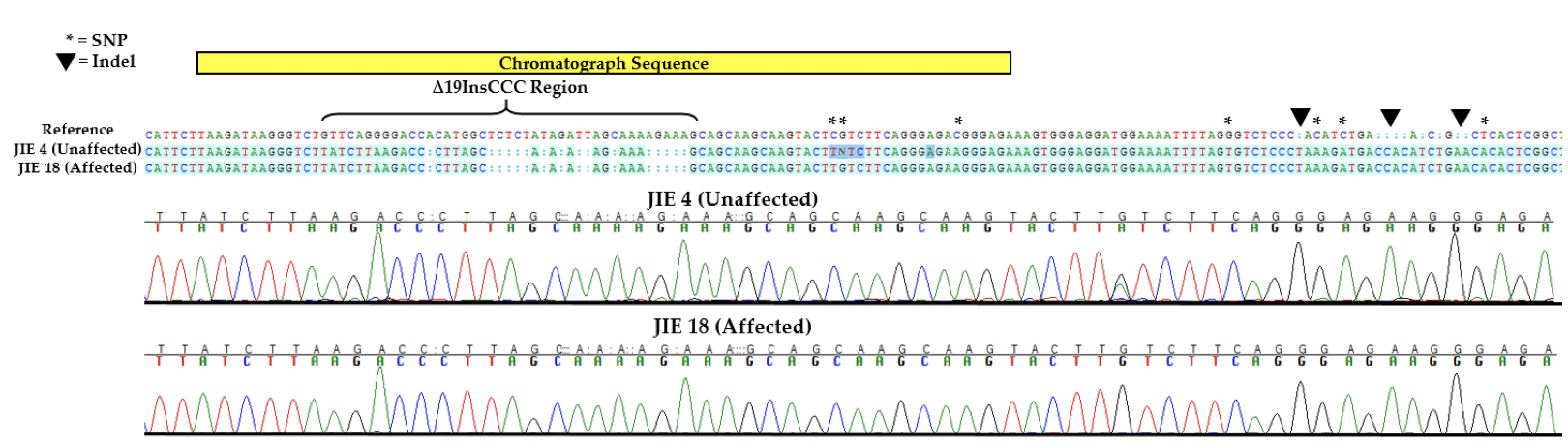

Supplement: Supplementary file 1 [file genes-10-00816-s001.zip › genes-610877 supplementary/genes-610877-supplementary.png]
